# Use of sugarcane–soybean intercropping in acid soil impacts the structure of the soil fungal community

**DOI:** 10.1038/s41598-018-32920-2

**Published:** 2018-09-27

**Authors:** Tengxiang Lian, Yinghui Mu, Qibin Ma, Yanbo Cheng, Rui Gao, Zhandong Cai, Bin Jiang, Hai Nian

**Affiliations:** 1 The State Key Laboratory for the Conservation and Utilization of Subtropical Agro-bioresources, South China Agricultural University, Guangzhou, 510642 Guangdong People’s Republic of China; 2 The Key Laboratory of Plant Molecular Breeding of Guangdong Province, College of Agriculture, South China Agricultural University, Guangzhou, 510642 Guangdong People’s Republic of China

## Abstract

Although sugarcane-soybean intercropping has been widely used to control disease and improve productivity in the field, the response of soil fungal communities to intercropping has not been fully understood. In this study, the rhizosphere fungal communities of sugarcane and soybean under monoculture and intercropping systems were investigated using Illumina MiSeq sequencing of ITS gene. Intercropping decreased the alpha-diversity and changed fungal community composition compared to monocultures. Taxonomic analyses showed that the dominant phyla were Ascomycota, Zygomycota and Basidiomycota. The abundance of Ascomycota decreased in intercropping sugarcane-grown soil compared to monoculture, while it increased in soybean-grown soil in the intercropping system. In addition, intercropping increased the abundance of important fungal genera, such as *Trichoderma*, *Hypocreales* and *Fusarium* but decreased the relative abundance of *Gibberella* and *Chaetomium*. The results of canonical correspondence analysis and automatic linear modelling indicated that fungal community compositions were closely associated with soil parameters such as total nitrogen (TN), soil organic matter (SOC), pH and NO_3_^−^, which suggests that the impacts of intercropping on the soil fungal community are linked to the alteration of soil chemical properties.

## Introduction

Worldwide, sugarcane-soybean intercropping was re-discovered as a sustainable and stable system when compared to its counterpart mono-system^1^. It has been recognized as a potential system for the augmentation of productivity over space and time in subsistence farming due to the high utilization efficiency of light, stability of yields, resilience to perturbations and reduction of N-leaching^2,3^. Many studies have shown that sugarcane can benefit from soybean in intercropped system because the highly efficient nitrogen fixation of soybean can improve soil fertility and field ecological conditions^4,5^. Soybean may also be impacted by intercropping since sugarcane absorbs more nitrogen and therefore stimulates legume’s biological nitrogen fixation^6^. In addition, greater amounts of potassium and phosphorus were taken up from intercropping soil than from the monoculture^3,6^.

In addition to the advantages described above, intercropping undoubtedly shifts the fungal community structure, which performs important ecological functions, including carbon (C) and nutrient cycling, plant-growth promotion, pathogenesis, and parasitism in agricultural ecosystems^7–9^. In an intercropping system, the roots of different plant species directly contact each other, and the fungal communities in rhizosphere of both plant species also interact with each other. Therefore, the fungal community is likely to be a mixture of the species-specific community that benefits plants^10^. In addition, root exudation enhanced rhizosphere fungal activity^11–13^, which can affect plant growth and fitness via hormone production or the mineralization of nutrient availability^14–16^.

With the development of high-throughput sequencing technologies, a comprehensive understanding of microbial community structure can be achieved, which will further reveal the impact of intercropping on microbial communities^17^. The effects of intercropping on the composition of rhizosphere microbial community are variable^18,19^, and the primary factors that cause the changes in microbial community are not clear. Previous studies have reported the response of soil fungal communities to intercropping in a broad range of plants, soils and ecosystems. For example, Zhou *et al*.^10^ used denaturing gradient gel electrophoresis (DGGE) analysis to investigate the effects of cucumber-onion on the diversity and structures of the fungal community in rhizosphere of cucumber and onion. They found that changes occurred in soil fungal populations as a response to different cropping systems. In addition, using the high-throughput sequencing technology, Rachid *et al*.^8^ studied soil fungal community diversity and structure in the Eucalyptus-*Acacia mangium* intercropping system. They found that intercropping *Acacia mangium* significantly increased the numbers of the soil fungal genera and the diversity indices and increased the frequency of several genera, such as *Pisolithus* and *Scleroderma*, that were not found in monoculture cultivation samples. In this study, we focused on change in fungal community structure in a sugarcane-soybean intercropping system.

The objectives of this study were to investigate the effects of intercropping on soil physicochemical properties and fungal community structure in an acid soil. In this study, we focused on analysing the soil fungal community by evaluating soil fungal abundance and community composition using quantitative real-time PCR (q-PCR) and Illumina MiSeq sequencing methods, respectively.

## Results

### Effect of intercropping on soil properties

Compared with monoculture, intercropping resulted in a decrease of rhizosphere pH from 6.63 to 6.10 and from 6.03 to 5.50 for sugarcane and soybean, respectively. Concentrations of organic matter, total nitrogen, phosphorus, potassium and NH_4_^+^-N in the rhizosphere significantly increased for the two plant species under intercropping compared with the monoculture (*p* < 0.05). Concentration of NO_3_^−^-N in rhizosphere of sugarcane and soybean under intercropping significantly decreased (*p* < 0.05) (Table 1).

**Table 1:** General chemical characteristics of the soil.

Treatment	SOC (g kg^−1^)	Total (g kg^−1^)			NH_4_^+^-N (mg kg^−1^)	NO_3_^−^-N (mg kg^−1^)	pH
		Nitrogen	Phosphorus	Potassium			
M-Sugarcane	16.53 ± 0.28	0.73 ± 0.06	0.58 ± 0.05	9.68 ± 0.29	5.56 ± 0.75	1.96 ± 0.15	6.63 ± 0.12
I-Sugarcane	17.25 ± 0.21	0.81 ± 0.01	0.67 ± 0.03	9.16 ± 0.24	8.01 ± 0.22	0.75 ± 0.63	6.1 ± 0.10
M-Soybean	16.67 ± 0.57	0.77 ± 0.02	0.59 ± 0.04	9.85 ± 0.42	6.43 ± 0.79	4.76 ± 0.85	6.03 ± 0.15
I-Soybean	16.95 ± 0.08	0.82 ± 0.03	0.68 ± 0.01	11.27 ± 0.61	11.59 ± 0.84	0.97 ± 0.56	5.5 ± 0.10
LSD	0.548	0.072	0.063	0.776	1.314	1.137	0.224

Values are the means ± SE (n = 3). M-Sugarcane, sugarcane monoculture; I-Sugarcane, intercropped sugarcane. M-Soybean, soybean monoculture; I-Soybean, intercropped soybean.

### Soil fungal abundance

**Figure 1:**
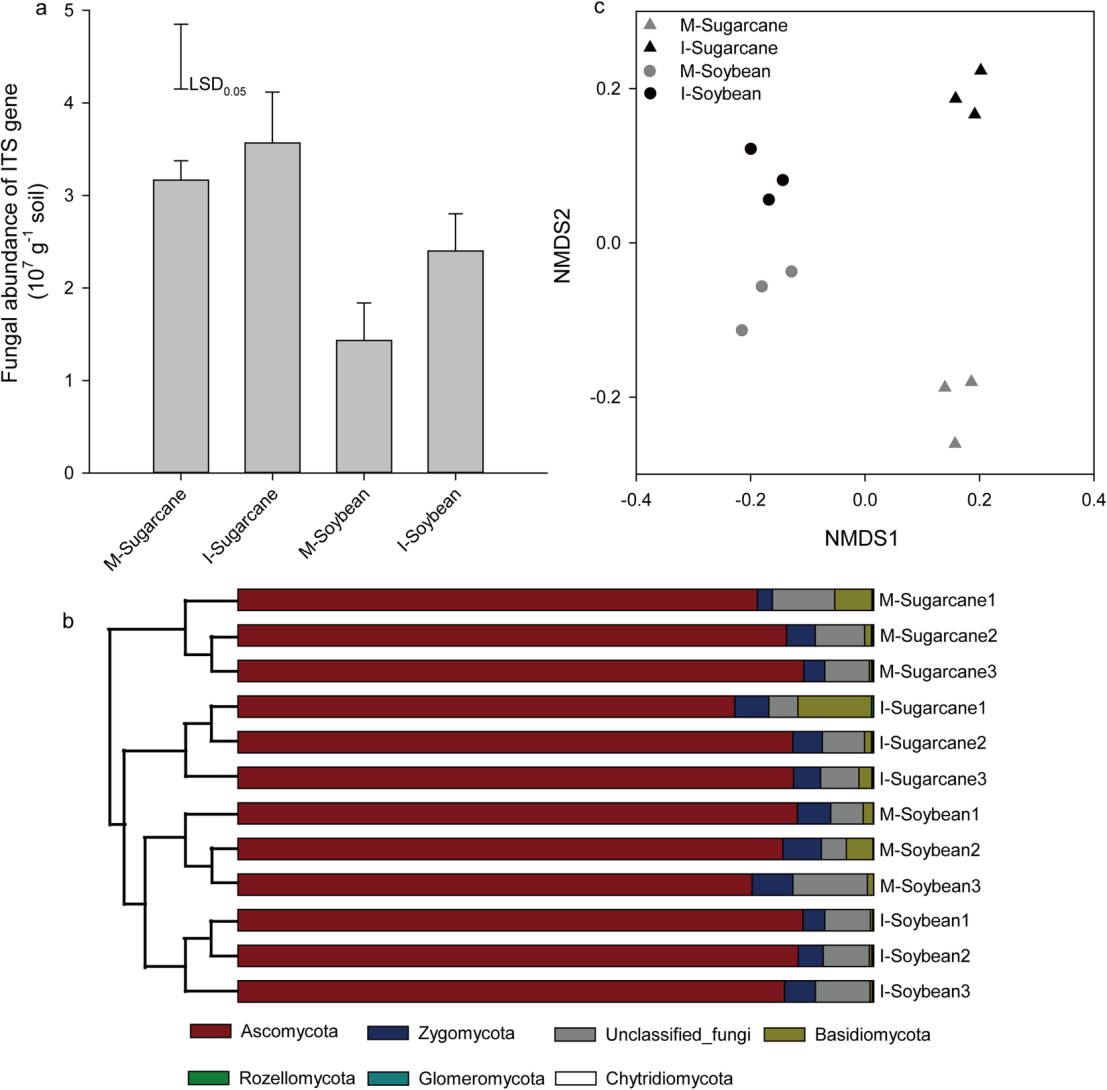
Comparison of the fungal communities in the soil on the basis of ITS gene analysis. (**a**) Fungal abundance was assessed using q-PCR, and data are the means and standard errors of 3 replicates. (**b**) Nonmetric multidimensional scaling (NMDS) plot of fungal communities in 12 soil samples under the three treatment regimes. (**c**) Phylogenetic relationships of fungal communities shown with the relative abundances of different fungal phyla. M-Sugarcane, sugarcane monoculture; I-Sugarcane, intercropped sugarcane. M-Soybean, soybean monoculture; I-Soybean, intercropped soybean.

The fungal abundances in all soil samples were determined using q-PCR targetting ITS1 gene. The abundance of fungi varied from 1.4 × 10^7^ gene copies g^−1^ dry soil to 3.57 × 10^7^ gene copies g^−1^ dry soil across all the samples (Fig. 1a). In general, soil fungal abundance significantly increased in soybean in the intercropping system but not in sugarcane. The fungal abundance was 1.7 times higher in intercropping soybean than that in the monoculture.

### Relative abundances of fungi at different taxonomic levels

**Figure 2:**
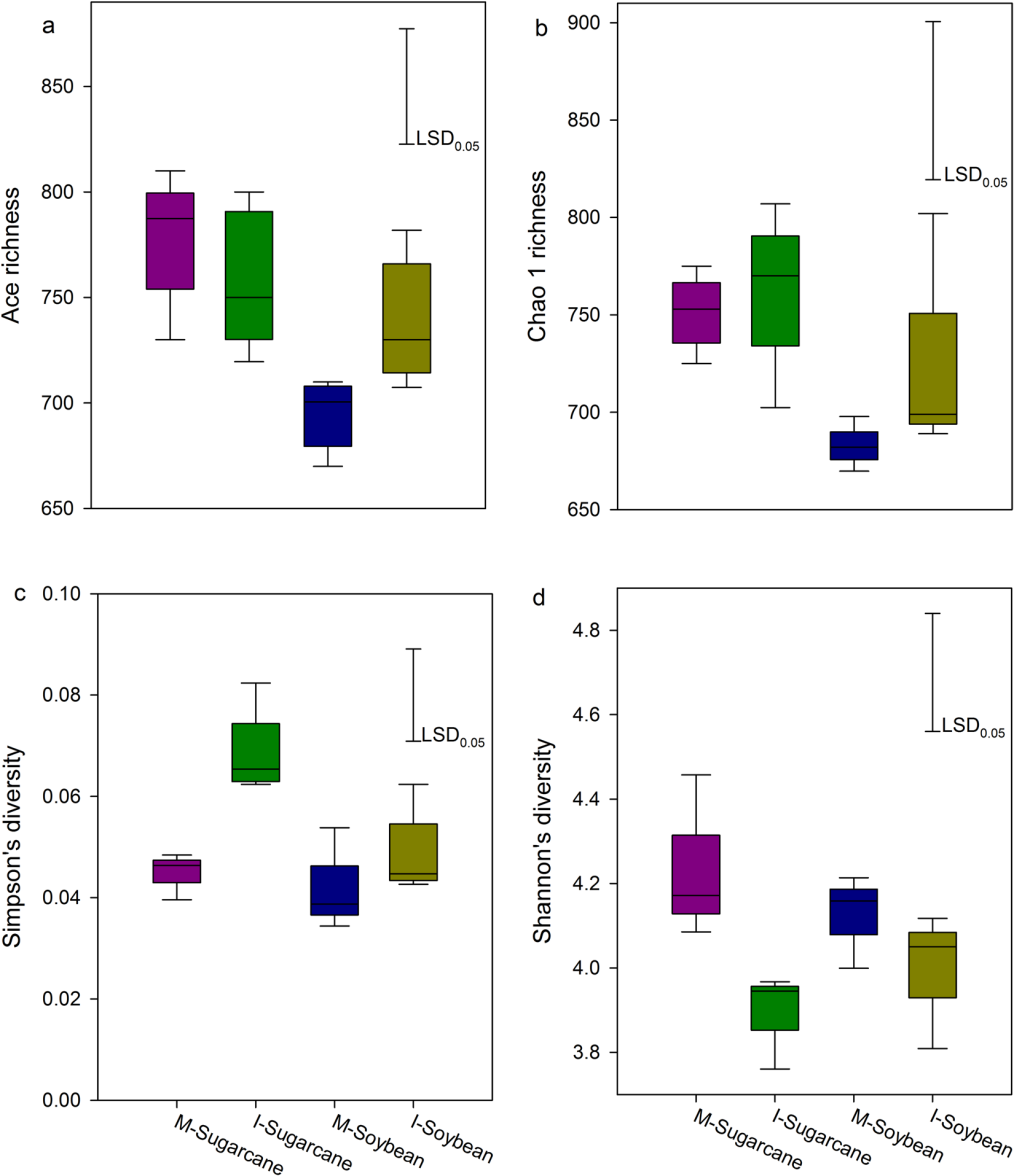
Illumina MiSeq sequencing of the fungal data and bacterial community diversity indices (at 97% sequence similarity) based on the ITS gene. (**a**) Ace richness (**b**) Chao 1 richness (**c**) Simpson’s diversity and (**d**) Shannon’s diversity. M-Sugarcane, sugarcane monoculture; I-Sugarcane, intercropped sugarcane. M-Soybean, soybean monoculture; I-Soybean, intercropped soybean.

In total, we obtained 356,712 quality sequences from all rhizosphere samples. The fungal diversity indices, including Ace richness (Fig. 2a), Chao 1 richness (Fig. 2b), Simpson’s diversity (Fig. 2c) and Shannon’s diversity (Fig. 2d) are shown. In general, a lower Shannon’s diversity index was found in rhizosphere of intercropped sugarcane compared to the monoculture (Fig. 2d). The dominant fungal phyla were Ascomycota, Zygomycota and Basidiomycota (Fig. 1b), and their relative abundances varied from 84.3% to 87.6%, 3.4% to 5.9% and 0.4% to 4.9%, respectively, across all the treatments (Fig. 1b, Table S1). Although the relative abundances of different fungal phyla fluctuated with intercropping treatments, no significant effects of intercropping treatments were observed with exception of the intercropping soybean, which showed that the relative abundance of Zygomycota was significantly decreased compared to the monoculture (Table S1).

Taxonomical classification at genus level revealed that more than 289 fungal genera or groups were detected in this study (data not shown). The most abundant genera primarily included *Fusarium* (20.49–24.98%), *unclassified_Chaetomiaceae* (1.55–7.50%), *unclassified_Sordariales* (2.05–5.38%), *Penicillium* (2.06–4.09%), *Gibberella* (1.36–6.40%), *unclassified_Nectriaceae (0.98–5.89%)*, *Trichoderma* (2.92–10.22%), *Chaetomium* (0.48–2.56%), *Gibellulopsis* (1.56–4.14%), *Dictyophora* (0.04–4.94%) and *Mortierella* (3.32–5.69%). Generally, the relative abundances of *Fusarium* and *unclassified_Chaetomiaceae* in Ascomycota significantly increased (Table S2), while the relative abundances of *unclassified_Sordariales* and *Gibberella* in Ascomycota, *Dictyophora* in Basidiomycota, and *Mortierella* in Zygomycota decreased under intercropping for both plant species compared to the monoculture (Table S2).

**Figure 3:**
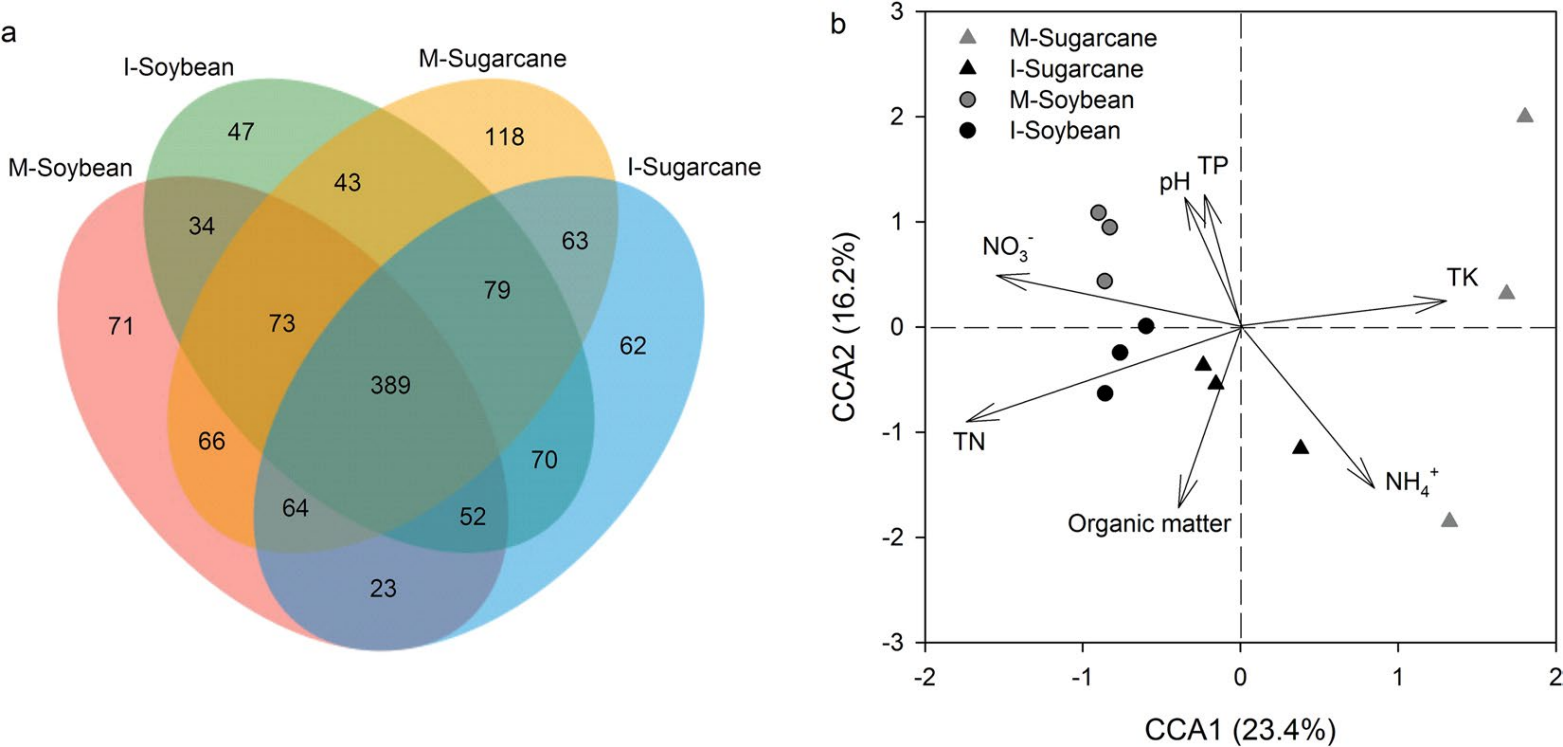
(**a**) Venn diagram showing the shared fungal OTUs in all soil samples. M-Sugarcane, sugarcane monoculture; I-Sugarcane, intercropped sugarcane. M-Soybean, soybean monoculture; I-Soybean, intercropped soybean, and (**b**) Canonical correspondence analysis (CCA) considering the relative abundance of fungal at the operational taxonomic unit (OTU) level and SOC, total N, P and K, concentrations of NH_4_^+^ and NO_3_^−^ and pH.

In addition, Venn diagrams revealed that the sum of total observed OTUs in the four treatments was 1255, which included 389 OTUs common to all treatments (Fig. 3a). The distribution of the sequences demonstrated once again that each plant rhizosphere had its own fungal populations.

### Overall structural changes in fungal communities

Nonmetric multidimensional scaling (NMDS) showed that fungal communities in rhizosphere of sugarcane under monoculture were clearly separated from the fungal communities under the intercropping system (Fig. 1c). A similar trend was found in soybean. Different plant species and cropping methods appeared to be the two primary factors in the first nonmetric multidimensional axis (NMDS 1) and the second nonmetric multidimensional axis (NMDS 2) (Fig. 1c).

### Relationships between fungal community structure and soil properties

**Figure 4:**
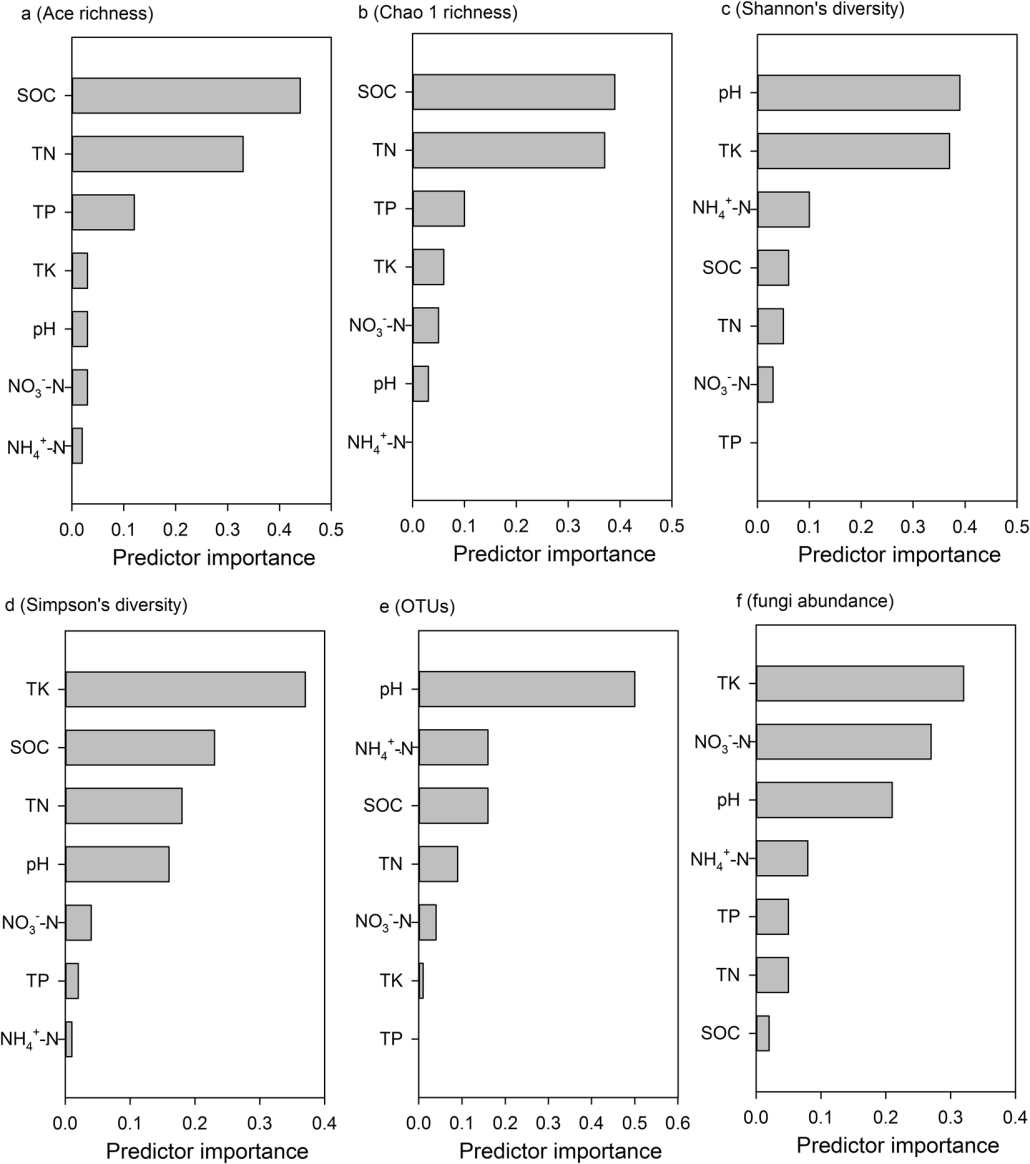
Predictive importance of each soil chemical property to (**a**) Ace richness, (**b**) Chao 1 richness, (**c**) Shannon’s diversity, (**d**) Simpson’s diversity, (**e**) the number of OTUs and, (**f**) fungal abundance determined by automatic linear modelling.

The results of Mantel test revealed positive or negative correlations between fungal community structure and following environmental factors listed based on Spearman’s correlation scores: TN, NH_4_^+^-N, TK, NO_3_^−^-N, SOC and TP (Table 2). The Shannon’s diversity index indicated that they were positively or negatively correlated with soil SOC and TP (Table 3). The results of automatic linear modelling revealed that SOC, soil pH and TK were the three most important predictors of the soil fungal diversity indices, OTU numbers and fungal abundance (Fig. 4). In addition, a canonical correspondence analysis (CCA) plot was displayed to compare the fungal community compositions among all soil samples and to identify the major environmental variables that affect community structure. Similar to the NMDS plot, the fungal communities shifted with plant species and intercropping along the CCA1 and CCA2, respectively. Among all the environmental variables tested, TN, TK and NO_3_^−^-N were relatively near CCA1, which explained 23.4% of the variation of the communities (Fig. 3b), indicating that these three variables play important roles in shifting fungal communities. In addition, SOC, soil pH and total P, which were near CCA2, had a role in shifting fungal communities in response to cultivation method along the CCA2, which accounted for 16.2% of the variation of the communities (Fig. 3b).

**Table 2:** The Spearman’s correlations (r) between the environmental variables and the fungal community structure (Bray-Curtis distance) determined by the Mantel test.

	r	*p*
TN	0.289	0.014
NH_4_^+^-N	0.183	0.092
TK	0.14	0.124
pH	0.129	0.147
NO_3_^−^-N	0.099	0.205
SOC	−0.006	0.503
TP	−0.037	0.584

**Table 3:** The Spearman’s correlations among the fungal community and soil properties.

Treatment	SOC	TN	TP	TK	NH_4_^+^-N	NO_3_^−^-N	pH
Ace richness	0.189	−0.476	−0.114	0.100	0.037	−0.272	0.229
Chao 1 richness	0.283	−0.344	−0.226	0.032	0.089	−0.126	0.156
Shannon’s diversity	−0.757**	−0.525	0.712**	0.177	−0.444	−0.225	0.465
Simpson’s diversity	0.675*	0.240	−0.700*	−0.463	0.153	0.376	−0.096
Fungal abundance	−0.311	−0.426	0.296	−0.207	−0.457	−0.163	0.605*
Fungal OTUs	0.256	−0.025	−0.301	−0.395	−0.070	0.060	0.355

## Discussion

Changes in soil fungal abundance as a result of intercropping have been an area of great concern^9^. In this study, soil fungal abundances increased in intercropping system (Fig. 1a), which is consistent with a previous study that showed that intercropping promoted soil fungal growth^19^. One of the possible explanations for the increasing fungal abundance observed in this study could be related to the greater quantity and types of root exudates in intercropping system^10^, and these exudates provide more energy and nutrition for fungi^10,12^. In addition, all changes in the soil physicochemical properties caused by intercropping in this study, such as the decrease of soil pH and the increase of soil nutrient contents (Table 1), provided substrates for microbial growth or improved micro-environments for microbial habitats^20^, which eventually resulted in an increase in the soil fungal abundance.

Previous reports have shown that soil pH is often the dominant factor in determining fungal community composition and diversity^21–24^. In addition, a large-scale investigation has also demonstrated that soil pH is a major factor in shifting fungal diversity^25^. This may be attributed to the changed pH impacting the pH homeostasis of microbial cells or regulating availability of soil nutrients^26^. In this study, soil pH in intercropped soybean soils decreased compared with monoculture soils. This finding suggests that soil pH drove fungal diversity in intercropping system. Our result is consistent with the results of Li *et al*.^27^, who stated that there was generally lower microbial diversity in intercropped soybean at low pH than that in the monoculture at high pH, although no statistical significance was detected.

Environmental factors may be directly related to the participation of certain taxa. The results of this study showed that SOC, soil pH and total N were the predominant factors in explaining fungal community structure (Fig. 4). Many studies have reported that intercropping alters soil properties, which could undoubtedly shift the microbial community structure^10,28–30^. Notably, SOC and pH have also been found to be the most important factors that determine microbial community structure in natural environmental systems, raising arguments on biogeographic patterns of microorganisms^23,31^. In addition, shifts in chemical properties of the total nitrogen could also potentially result in changes in microbial community structure for reasons of resource competition^32–34^.

Legumes can fix atmospheric N_2_ in symbiosis with *Rhizobium* and complement non-legumes in the intercropping system^28^. Intercropped sugarcane acquired higher N than that in monoculture system. Most studies showed that N_2_ fixation efficiency of legumes could be improved when intercropped with non-legumes^35,36^. Intercropping of soybean with sugarcane improved soil TN and SOC due to the organic matter inputs in the form of litterfall and fine roots from the soybean and sugarcane, indicating the important plant-soil feedback process. The NMDS analysis clearly demonstrated that the fungal communities separated into four groups based on plant species and cultivation mode, suggesting that plant species and cropping method appeared to be the two primary factors determining the fungal community in the acid soils (Fig. 1c). Previous studies also observed that intercropping influences are mediated by a general stimulation of soil microorganisms^9,37^. This may be due to roots of different plant species directly contacting each other, which results in the interaction of root exudates from both plant species and therefore changes the habitat for soil fungi^10^.

The fungal community showed differences among intercropping and monoculture treatments in the clustering tree analysis (Fig. 1b), which were consistent with the results for microbial abundance and diversity. Some fungal genera changed significantly in intercropping sugarcane (e.g., *Trichoderma* and *unclassified_Chaetomiaceae*) and intercropping soybean (e.g., *unclassified_Hypocreales* and *Chaetomium*) treatments, which indicated that intercropping had a significant effect on certain fungal genera (Table S2). The increased *Trichoderma* in intercropped sugarcane may control a wide range of phytopathogens^38^ because *Trichoderma* secretes chitinases and cellulases, which can hydrolyse pathogen cell walls^39^. In addition, *Hypocreales* was reported to be a mortality agent against European corn borer *Ostrinia nubilalis* in maize^40^. In this study, the increased relative abundance of *Hypocreales* in intercropped soybean may reduce pests in the field. However, some genera, such as *Chaetomium*, were decreased in intercropped system of both sugarcane and soybean. *Chaetomium* appears to play a multifunctional role, such as serving as a soil cellulose degrader, biocontrol agent and saprophyte that becomes parasitic under severely C-limiting conditions^41^.

One of the most noteworthy findings in this study was the relative abundances of *Fusarium* (Table S2) in intercropping sugarcane and intercropping soybean. Some species of *Fusarium*, such as *F. graminearum, F. virguliforme*, *F. proliferatum*, *F. sporotrichioides*, and *F. solani*, are soybean pathogens causing soybean root rot^40^. Notably, plants release enormous amounts of chemicals through their roots, at a significant carbon cost, to combat pathogenic microorganisms and attract beneficial ones^42^. Our findings suggest that intercropping may negatively increase the influence of *Fusarium* on crop growth. Although some species of *Fusarium* are pathogenic to plants, there are many non-pathogenic species of *Fusarium*. A higher abundance of *Fusarium*, such as *F. graminearum*, indicates that there are more decomposers^43^. In addition, *Fusarium* species can use many forms of nitrogen and could mediate the effects of fertilizers via increased plant vigour^44^. In contrast, the relative abundance of another pathogenic genus, *Gibberella*, was significantly decreased in intercropping treatments (Table S2). *Gibberella* is a well-known pathogen that has been implicated in the diseases of several agricultural crops, including rice, sugarcane and maize^45–47^.

## Conclusions

Sugarcane–soybean intercropping in acid soil altered soil properties, decreased fungal diversity and changed the fungal community structure. In particular, intercropping influenced the relative abundances of some soil-borne plant pathogens, such as *Fusarium* and *Gibberella*. CCA analysis and automatic linear modelling revealed that changes in the soil fungal community composition were related to the soil characteristics, including SOC, TN, pH and total phosphorus, suggesting that the effects of intercropping on soil fungal community were indirectly driven through changes in the soil properties. In addition, this study was conducted on acidic soil, and diversity profiling was observed at an early stage of plant development. Future research should explore the effects of this intercropping in different soil types and plant stages.

## Materials and Methods

### Plant materials and experimental design

This study used soybean cultivar HuaChun 5 and sugarcane cultivar ROC 22, which are widely grown in South China. Plants were grown in pots in the glasshouse at South China Agriculture University, Guangzhou, China. The experiment had a random block design comprising three treatments with three replications: (1) sugarcane monoculture, (2) soybean monoculture and (3) soybean intercropped with sugarcane. Each pot contained 30 kg of a sieved soil, classified as an Ali-Udic Argosol, at pH 5.1, SOC 8.5 g kg^−1^, 0.41 g kg^−1^ N and 0.42 g kg^−1^ P. Two sugarcane seedlings or three soybean seeds were planted in a pot under the monoculture system, or two sugarcane seedlings with three soybean seeds were planted under intercropping system. The row spacing was 0.9 m for sugarcane and 0.3 m for soybean in all treatments, and rhizosphere soils from plants in the same pot were collected separately, and contact with each other was avoided. The soil water content was maintained at 80 ± 5% of field water capacity. Plants were harvested at the flowering stage.

### Soil sampling and measurements

Rhizosphere soil was recovered on 25 May 2016 (40 days after sowing) by gently shaking the soil from around the roots into a polyethylene bag and then mixing thoroughly. Approximately 2 g of each soil sample was placed in an autoclaved microcentrifuge tube (2 mL) and stored at −80 °C for DNA extraction. The remaining soil was air-dried at room temperature to measure soil chemical properties.

The soil pH was determined in a soil water suspension (1:5 w/v) using a pH meter. Soil total nitrogen content was measured using an Elemental Analyser (VarioEL III, Germany). Soil total phosphorus, ammonium nitrogen (NH_4_^+^-N) and nitrate nitrogen (NO_3_^−^-N) were assayed using a continuous flow analytical system (SKALAR SAN++, The Netherlands). Soil total potassium was quantified using inductively coupled plasma-atomic emission spectrometry (ICPS-7500, Shimadzu, Japan). Organic matter was measured using the potassium dichromate oxidation method previously described by Liu^48^.

### Soil DNA extraction and quantitative real-time PCR (q-PCR)

DNA was extracted using a Fast DNA SPIN Kit for Soil (Qbiogene Inc., Carlsbad, CA, USA) according to the manufacturer’s instructions. Quantitative PCR (q-PCR) was conducted by targeting the fungal ITS1 gene, using the ITS1 and ITS2 primers as described by White *et al*.^49^. The abundance of the fungal ITS gene was calculated using a regression equation to convert the cycle threshold (Ct) value to the known number of copies in the standard curves.

### Illumina MiSeq sequencing analysis

For sequencing, the primers ITS1F/ITS2R with 12 nt unique barcodes were used to amplify the fungal ITS. The following thermal programme was used for PCR amplification: 94 °C for 3 min, followed by 35 cycles at 94 °C for 30 s, 55 °C for 30 s, and 72 °C for 30 s, followed by an extension at 72 °C for 10 min^50^. Equimolar amounts of the PCR products were pooled and prepared for sequencing using the MiSeq sequencer^51^. The raw sequences were processed and analysed using QIIME Pipeline Version 1.8.0 (http://qiime.org/). Low-quality sequences shorter than 200 bp in length and with an average quality score of less than 20 were excluded from further analysis. High-quality sequences were clustered into operational taxonomic units (OTUs) at 97% sequence similarity using CD-HIT^52^. The OTUs were analysed using the UNITE database^53^, and those with more than 80% sequence similarity were preserved. A representative sequence of the OTUs was aligned using the Python Nearest Alignment Space Termination (PyNAST)^54^ with a phylogenetic tree built using Fast Tree^55^. The taxonomy of each representative phylotype was assigned using a BLAST comparison against sequences within the GenBank database. All of the sequences were deposited in the GenBank Sequence Read Archive (SRP129902).

### Statistical analysis

UniFrac statistical analysis was performed online at http://bmf.colorado.edu/unifrac/ to provide the index of community distance between each pair of samples^56^. A nonmetric multidimensional (NMDS) analysis was performed to indicate patterns of similarity (Bray-Curtis similarity) in the structure of the microbial community between treatments^57^. A canonical correspondence analysis (CCA) was conducted to explore the association of fungal community composition with soil characteristics. The NMDS and CCA analyses were performed using the “vegan” package in R version 3.2.0 for Windows^58^. Shannon’s diversity index, Simpson’s diversity index, Chao 1 richness and Ace richness were calculated in QIIME and used to compare the soil fungal alpha diversity. Venn diagrams of unique and shared OTUs were drawn in order to highlight the similarities and shared sequences between the different samples analysed. The Spearman’s correlation coefficients were calculated using the IBM Statistical Product and Service Solutions (SPSS) Statistics for Windows (Version 24), and the results were subject to a *t*-test for significance. Automatic linear modelling was performed at the confidence level of 95% in IBM SPSS Statistics for Windows.

An analysis of variance (ANOVA)^59^ was performed using Genstat 13 (VSN International, Hemel Hemspstead, UK) to assess the effect of treatments on SOC, total N, P and K, concentrations of NH_4_^+^-N and NO_3_^−^-N, pH and the relative abundance of fungal groups at genus and OTU levels. We only show the genera that had a significant response (*p* < 0.05) to the treatments. This was based on the least significant difference (LSD) at the significance level of *p* < 0.05.

## Electronic supplementary material

Table S1 2
