# Supplementary material for: Use of sugarcane–soybean intercropping in acid soil impacts the structure of the soil fungal community
Source: Sci Rep. 2018 Sep 27;8:14488. doi: 10.1038/s41598-018-32920-2 (PMC6160455; doi:10.1038/s41598-018-32920-2)
Supplement: Supplementary file 1 — Table S1 2 [file 41598_2018_32920_MOESM1_ESM.pdf]

Title page

Title: Use of sugarcane–soybean intercropping in acid soil impacts the structure of the soil fungal community

Tengxiang Lian[1,2], Yinghui Mu[1,2], Qibin Ma[1,2], Yanbo Cheng[1,2], Rui Gao[1,2], Zhandong Cai[1,2], Bin Jiang[1,2], Hai Nian[1,2]*

1.The State Key Laboratory for Conservation and Utilization of Subtropical Agro-bioresources, South China Agricultural University, Guangzhou 510642, Guangdong, People’s Republic of China

2.The Key Laboratory of Plant Molecular Breeding of Guangdong Province, College of Agriculture, South China Agricultural University, Guangzhou 510642, Guangdong, People’s Republic of China

[*] **Corresponding author:** Hai Nian

Corresponding address: No.483 Wushan Road, Guangzhou, Guangdong, 510642, China.

Tel: +8602085288024

Fax: +860208528802

E-mail address: hnian@scau.edu.cn

Table S1 Cultivation mode, crop species and their interactive effects on phylum abundance. *p* values less than 0.05 are indicated in bold letters. I-Sugarcane, intercropped sugarcane. M-Sugarcane, sugarcane monoculture; I-Sugarcane, intercropped sugarcane. M-Soybean, soybean monoculture; I-Soybean, intercropped soybean.

	Treatment							
	Relative abundances (%)				ANOVA (*p*-values)			
Phylum	M-Soybean	I-Soybean	M-Sugarcane	I-Sugarcane	Culture mode	Crop species	Culture mode* Crop species	LSD
Ascomycota	84.8 ±3.64	87.7 ±1.51	85.7 ±3.69	84.28 ±5.3	0.754	0.567	0.368	7.12
Zygomycota	5.9 ±0.61	4.07 ±0.73	3.41 ±1.08	4.75 ±0.54	0.594	0.074	**0.007**	1.445
unclassified_fungi	6.91 ±4.2	7.66 ±0.8	8.2 ±1.43	5.76 ±1.06	0.544	0.824	0.267	4.357
Basidiomycota	2.26 ±1.7	0.39 ±0.02	2.43 ±2.99	4.9 ±5.81	0.88	0.263	0.299	6.355
Rozellomycota	0.01 ±0.01	0.04 ±0	0.1 ±0.02	0 ±0	**<0.001**	**0.006**	**<0.001**	0.024
Glomeromycota	0.02 ±0.02	0.1 ±0.04	0.18 ±0.03	0.26 ±0.05	**0.004**	**<0.001**	0.891	0.064
Chytridiomycota	0.02 ±0.03	0.06 ±0.03	0.01 ±0	0.05 ±0.04	0.056	0.555	0.875	0.056

Table S2. Cultivation module, crop species and their interactive effects on genus abundance (read numbers in each genus) of fungal at phylum and genus levels. *p* values less than 0.05 are indicated in bold letters. M-Sugarcane, sugarcane monoculture; I-Sugarcane, intercropped sugarcane. M-Soybean, soybean monoculture; I-Soybean, intercropped soybean.

		Treatment<br>Relative abundances (%)				ANOVA (*p*-values)			
Phylum	Genus	M-Sugarcane	I-Sugarcane	M-Soybean	I-Soybean	Culture mode	Crop species	Culture mode* Crop species	LSD
Ascomycota	Fusarium	20.49±1.6	24.68±1.98	21.93±2.92	24.98±2.31	0.147	0.711	0.807	7.35
Ascomycota	unclassified_ Chaetomiaceae	1.55±0.24	3.63±0.45	5.21±0.55	7.5±1.03	**0.009**	**<0.001**	0.878	1.18
Ascomycota	unclassified_ Sordariales	5.38±1.4	2.05±0.55	4.86±0.19	2.2±0.22	**0.004**	0.812	0.678	0.947
Ascomycota	Penicillium	2.06±0.47	2.9±0.24	4.09±0.17	3.75±0.46	0.508	**0.004**	0.141	1.297
Ascomycota	Gibberella	1.98±0.34	1.36±0.09	3.4±0.22	2.51±0.4	**0.032**	**0.002**	0.673	1.344
Ascomycota	unclassified_ Nectriaceae	3.6±0.34	2.98±0.26	3.22±0.08	5.89±0.66	**0.032**	**0.013**	**0.003**	0.962
Ascomycota	Trichoderma	2.97±0.21	10.22±0.74	2.92±0.12	2.58±0.27	**<0.001**	**<0.001**	**<0.001**	0.524
Ascomycota	Chaetomium	0.5±0.08	0.48±0.04	2.56±0.3	0.49±0.06	**<0.001**	**<0.001**	**<0.001**	0.388
Ascomycota	Gibellulopsis	1.56±0.13	1.68±0.04	2.53±0.05	4.14±0.13	**<0.001**	**<0.001**	**<0.001**	1.957
Ascomycota	unclassified_ Sordariomycetes	2.84±0.5	4.64±1.02	2.42±0.33	1.7±0.17	0.394	**0.023**	**0.069**	0.569
Ascomycota	Phialemonium	1.53±0.13	0.59±0.06	2.19±0.08	0.58±0.03	**<0.001**	**0.004**	**0.003**	0.837

Ascomycota	Acremonium	1.37±0.31	0.58±0.03	2.03±0.16	0.8±0.04	**<0.001**	**0.037**	0.241	0.412
Ascomycota	Corallomycetella	2.77±0.48	0.59±0.07	1.59±0.14	0.81±0.08	**<0.001**	0.098	**0.026**	1.606
Ascomycota	Myrothecium	1.3±0.12	0.65±0.1	1.79±0.19	0.38±0.06	**<0.001**	0.412	**0.016**	0.668
Ascomycota	unclassified_ Pleosporales	1.38±0.2	0.93±0.08	1±0.14	1.66±0.32	0.626	0.417	**0.026**	0.552
Ascomycota	Clonostachys	0.54±0.05	1.22±0.25	1.5±0.16	1.34±0.15	0.167	**0.013**	**0.038**	1.876
Ascomycota	Monographella	0.57±0.24	0.53±0.07	1.22±0.09	2.8±1.12	0.215	**0.035**	0.198	0.645
Ascomycota	Roussoella	0.2±0.02	0.89±0.04	1.11±0.11	0.55±0.02	0.339	**0.002**	**<0.001**	0.332
Ascomycota	Colletotrichum	0.09±0.02	0.2±0.03	0.83±0.04	1.81±0.2	**<0.001**	**<0.001**	**0.003**	0.239
Ascomycota	Humicola	1.53±0.1	0.77±0.06	0.54±0.06	0.83±0.07	**0.012**	**<0.001**	**<0.001**	0.250
Ascomycota	Metarhizium	0.23±0.01	0.46±0.04	0.64±0.03	0.56±0.14	0.369	**0.011**	0.078	0.116
Ascomycota	Myrmecridium	0.34±0.04	0.2±0.01	0.63±0.06	0.08±0.01	**<0.001**	**0.036**	**<0.001**	0.827
Ascomycota	Talaromyces	0.33±0.01	1.37±0.43	0.9±0.21	0.64±0.17	0.161	0.773	**0.034**	0.096
Ascomycota	Lectera	0.01±0	0.04±0.01	0.52±0.06	0.41±0.02	0.265	**<0.001**	**0.037**	0.224
Ascomycota	Purpureocillium	0.22±0.04	0.16±0.06	0.59±0.11	0.16±0.03	**0.007**	**0.026**	**0.032**	0.324
Ascomycota	unclassified_ Hypocreales	0.42±0.03	1.06±0.1	0.47±0.01	2.31±0.17	**<0.001**	**<0.001**	**<0.001**	0.206
Ascomycota	Zopfiella	0.42±0.07	0.52±0.05	0.27±0.08	0.19±0.04	0.904	**0.005**	0.209	2.127
Ascomycota	Taeniolella	5.42±1.29	1.5±0.14	0.24±0.08	1.08±0.12	**0.046**	**0.003**	**0.007**	0.132
Ascomycota	Sagenomella	0.28±0.05	0.27±0.04	0.44±0.05	0.18±0	**0.011**	0.335	**0.016**	0.215
Ascomycota	unclassified_ Bionectriaceae	0.71±0.11	0.21±0.02	0.45±0.07	0.39±0.01	**0.003**	0.584	**0.009**	0.327
Ascomycota	Chaetosphaeria	0.13±0.02	0.31±0.04	0.34±0.02	0.19±0.03	0.73	0.176	**<0.001**	0.102
Ascomycota	Westerdykella	0.41±0.03	0.16±0.01	0.28±0.05	0.24±0.01	**0.002**	0.494	**0.01**	0.097
Ascomycota	unclassified_ Trichocomaceae	0.16±0.02	0.33±0.04	0.28±0.04	0.39±0.01	**0.001**	**0.014**	0.35	0.096

Ascomycota	Phialosimplex	0.34±0.04	0.09±0.02	0.2±0.01	0.11±0.03	**<0.001**	0.083	**0.032**	0.281
Ascomycota	Pyrenochaetopsis	0.61±0.16	0.45±0.05	0.22±0.01	0.15±0.01	0.214	**0.004**	0.656	0.167
Ascomycota	Curvularia	0.38±0.09	0.14±0.03	0.25±0.03	0.05±0.01	**0.003**	0.067	0.758	3.813
Ascomycota	Xylomyces	0.45±0.06	0.28±0.02	0.21±0.02	0.26±0.07	0.27	**0.025**	**0.047**	3.193
Ascomycota	Chloridium	0.35±0.03	0.66±0.06	0.13±0.02	0.22±0.03	**<0.001**	**<0.001**	**0.015**	0.035
Ascomycota	Cordyceps	0±0	0.03±0	0.06±0.01	0.52±0.02	**<0.001**	**<0.001**	**<0.001**	0.088
Ascomycota	Volutella	0±0	0.08±0.02	0.09±0.02	1.21±0.02	**<0.001**	**<0.001**	**<0.001**	0.555
Ascomycota	Paraphaeosphaeria	1.44±0.28	1.6±0.19	0.07±0.02	0.18±0.04	0.441	**<0.001**	0.893	1.001
Basidiomycota	Dictyophora	0.04±0.01	0.05±0.01	1.94±0.99	0.04±0	0.089	0.091	0.088	1.392
Zygomycota	Mortierella	4.5±0.31	3.32±0.6	5.68±0.34	3.93±0.4	**0.009**	0.068	0.516	2.083
